# A rodent model of enhanced anticipation of positive events: sex-specific modifications in cognitive bias and emotional resilience

**DOI:** 10.3389/fnbeh.2025.1643979

**Published:** 2025-11-13

**Authors:** Sarah C. Hartvigsen, Megan Hooper, Olivia Harding, Evelyn Barringer, Isabel DiLandro, Aditya Narayanan, Brendan Crockett, Yulia Shatalov, Isabella Tomé, Paean Luby, Braden Wixted, Molly Kent, Kelly Lambert

**Affiliations:** 1 Department of Psychology, University of Richmond, Richmond, VA, United States; 2 Department of Biology, Virginia Military Institute, Lexington, VA, United States

**Keywords:** anticipation, cognitive bias, sex differences, positive events, emotional regulation

## Abstract

While it is known that chronic unpredictable stress and negative events adversely affect neurobiological outcomes, much less is known regarding the neurobiological impact of positive emotions such as chronic anticipation of appetitive events. From a translational perspective, an enhanced understanding of the impact of extended exposure to positive emotions may provide novel insights into effective non-pharmacological, behavior-based approaches to enhance mental resilience. Here, we investigate a novel rodent model of chronic Unpredictable Positive Event Response (UPER) training in male and female Long Evans rats to examine behavioral, neural, and endocrine effects of enhanced anticipation of positive events. Rats were exposed to either 3 weeks of daily, randomly administered, cued positive events (UPER training) or exposure to the same positive events administered at the same time (i.e., in a predictable manner) each day to control for anticipation (Enriched Control Training; ENR). Following UPER and ENR training, rats were assessed for cognitive bias, exploratory behaviors, and persistence in a Cognitive Bias Assessment paradigm, Novelty-Suppressed Feeding Task, and an Unattainable Puzzle Reward Task, respectively. In the Cognitive Bias Assessment, a trend for UPER-trained males to respond with an optimistic bias was observed. A main effect of training was observed in the Unattainable Puzzle Reward Task, with UPER-trained rats exhibiting reduced latency to interact with the novel object. A sex-dependent latency to consume a food reward in a Novelty-Suppressed Feeding Task was also seen. Focusing on fecal corticosterone metabolite (FCM) levels following anticipation-enhanced versus anticipation-minimized training, UPER-trained rats exhibited a trend for lower levels than ENR-trained rats. No c-fos activation differences were observed between the groups. Overall, these preliminary findings suggest that anticipation for positive events may have sex-specific effects on emotional responses to uncertain events. Accordingly, further research may determine relevance of this model in preclinical models of psychiatric diseases.

## 1 Introduction

Major depressive disorder (MDD), affecting over 320 million people worldwide, is a leading cause of disability-adjusted life-years (World Health Organization, 2017; Santomauro et al., 2021). Unfortunately, the COVID-19 pandemic exacerbated the global burden of MDD, as cases are estimated to have accelerated by nearly 30% (Vos et al., 2020; Wang et al., 2025). Individuals with MDD often present with emotional symptoms, including depressed mood and motivation, anhedonia, hopelessness, and excessive feelings of worthlessness. Examples of cognitive symptoms include deficits in executive function, concentration, memory, and adaptive decision-making, as well as negative cognitive bias (for a review see Hammar et al., 2022; Keller et al., 2019; Malhi and Mann, 2018; Nuño et al., 2021). Current pharmaceutic-focused outcomes for these emotional and cognitive symptoms of depression often have unwanted side effects and low efficacy rates, with the most marked improvements observed in severely depressed patients (Braund et al., 2021; Kirsch et al., 2008; Rush et al., 2006). This limited efficacy of current depression treatments underscores the need for novel interventions targeting both emotional and cognitive symptoms.

This gap in effective interventions highlights the importance of exploring non-pharmacological approaches to enhance mental resilience. Behavior-based treatments and interventions such as Cognitive Behavioral Therapy (Porto et al., 2009) drive changes in brain functioning and circuitry to confer mental resiliency and improve symptoms of mental illness via the modification of relevant neural functions. Accordingly, our lab introduced the term behaviorceuticals to refer to intentional behavioral interventions designed to modulate neurochemicals and enhance mental health, offering a complementary approach to traditional pharmacological treatments (Bardi et al., 2012). One potential behavioral intervention that is accessible and affordable is the anticipation of positive events. In support of behavioral and experiential therapeutic interventions, previous research suggests that positive life events such as experiencing an enjoyable event, monetary increase, or desired social contact are associated with the emergence of less-severe depressive symptoms and more positive life events (Disabato et al., 2017; Hovenkamp-Hermelink et al., 2019; Lewinsohn and Graf, 1973; Lewinsohn and Libet, 1972; Spinhoven et al., 2011).

Although the neurobiological effects of positive emotional experiences have received less attention than neurobiological effects of negative emotional experiences, research suggests that positive events play an important role in emotional regulation; additionally, the expectation of potential future positive events also influences emotional health (Monfort et al., 2015; Rief and Joormann, 2019). Whereas healthy individuals are more likely to update future expectations in a more positive manner after experiencing positive events and feedback compared to updating negative expectations after receiving negative events and feedback, individuals with depression do not display this optimistically-weighted updating (Hobbs et al., 2022; Hoffmann et al., 2024; Korn et al., 2014; Kube et al., 2020). This “expectation-focused model of depression” posits that MDD is often characterized by more anticipation of negative future events than anticipation of positive future events as well as the inability to reappraise the future more positively (Kube et al., 2020).

Anticipation of positive rewards is also associated with optimistic cognitive strategies. Specifically, anticipating positive events and rewards in the face of uncertainly is consistent with optimism, a cognitive response associated with adaptive health outcomes and resiliency following stress exposure, likely due to the integration of cognitive and stress response neural circuits leading to a buffering of the stress-related increase in cortisol (Fredrickson et al., 2009; Hu and Yang, 2021; Jobin et al., 2014; Kube et al., 2020; Leslie-Miller et al., 2021). Depressed individuals have been found to maintain a less optimistic cognitive style than healthy individuals and are often unable to update this style to become more optimistic despite occurrence of events that increase the probability of a future positive event (Hobbs et al., 2022; Kube et al., 2018). Consequently, it is important to identify potential therapeutic approaches that facilitate the transition to increased optimistic cognitive styles prior to the emergence of depressive symptoms, a process termed “cognitive immunization” (Kube et al., 2019; Rief et al., 2015). Alternatively, because negative cognitive bias interacts with negative events to increase susceptibility to pessimistic cognitive strategies and increase susceptibility to depression (Haeffel and Vargas, 2011), it is critical to determine if an individual’s cognitive bias and associated neural circuits can be reshaped during periods of health *prior* to exposure to stressful life events in a manner that confers resiliency against the onset of depressive symptoms. Thus, identifying ways to reshape pessimistic and optimistic cognitive strategies could offer protective benefits against future depressive symptoms (Haeffel and Vargas, 2011).

Given the need for empirically-driven interventions to shift cognitive strategies in an optimistic manner, the purpose of the current study was to examine how chronic exposure to positive events, specifically *enhanced anticipation* of those positive events, may reshape cognitive bias, promote resiliency-related behaviors and modulate stress hormone levels. To examine this question, we developed a rodent model of chronic, enhanced expectation of positive events (i.e., Unpredictable Positive Event Response; UPERs). Given that positive events have been shown to influence the remission of depressive symptoms as well as improve positive emotion (Disabato et al., 2017; Hovenkamp-Hermelink et al., 2019; Lewinsohn and Graf, 1973; Lewinsohn and Libet, 1972; Spinhoven et al., 2011), we hypothesized that chronic enhanced expectation of positive event training in male and female rats would be associated with increased optimistic cognitive bias, elevated persistence in a problem-solving task and enhanced exploratory behaviors. Further, we hypothesized that UPER-trained rats would display modified endocrine and neural markers of stress compared to their enriched control (ENR) counterparts. Given the success of behavioral strategies such as CBT, the proposed behavioral training program provides opportunities to identify anticipation-induced changes to cognitive bias in health, with the potential for future investigations to utilize this rodent model of enhanced anticipation in preclinical studies of psychiatric conditions.

## 2 Methods

### 2.1 Animals

Twelve male and twelve female Long Evans outbred rats (*n* = 6 per group) weighing 75–99 g (~4 weeks old) on arrival were ordered from Envigo (Indianapolis, Indiana). Upon arrival, rats were randomly assigned to standard home cages with aspen bedding and group-housed with 3 per cage to habituate to the lab for 1 week. Rats were given *ad libitum* access to standard chow diet (Teklad Global Diet 2018, Inotiv, West Lafayette, IN, USA) and water and kept on a 12-h light/dark schedule. All rats were handled and given one piece of Froot Loops® cereal (Kellogg Company, Battle Creek MI, USA) and one piece of Cheerios® cereal (General Mills, Inc., Minneapolis, MN, USA) daily. On the last day of habituation, a baseline fecal sample was taken at 9:00 a.m. before rats were transferred from their standard home cage to a larger enriched cage that was equipped with a small wooden structure and a paper towel for nesting material; all animals remained in their new enriched cages with their originally assigned standard cage mates. Throughout the study, all rats were treated in compliance with, and all protocols approved by, the University of Richmond’s Institutional Animal Care and Use Committee.

### 2.2 Cued unpredictable positive event response (UPER) and enriched control (ENR) training

**Figure 1:**
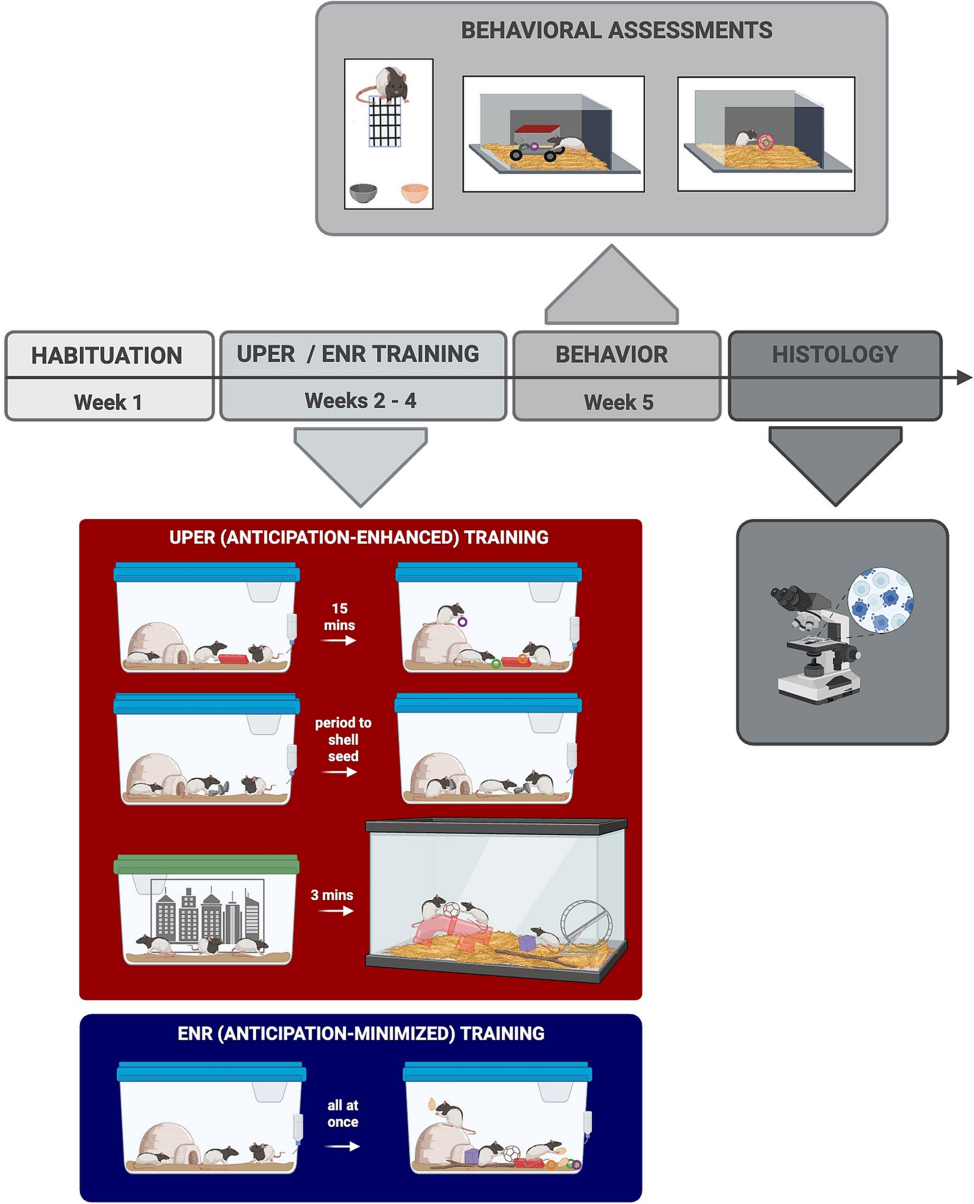
Experimental timeline. Following 1 week of habituation, rats were exposed to either enriched positive event training with anticipation or enriched control training with minimized anticipation of positive events. Rats that were randomly allocated to the enhanced anticipation group received enriched Unpredictable Positive Event Response (UPER) training, which consisted of three cued positive events daily given at randomized times throughout the day. The three randomly administered cued events were (1) a Lego® block placed in home cage for 15 min followed by Froot Loops®, (2) sunflower seeds with the shell intact, and (3) transfer of rats to a different context (transfer cage) for 3 min after which they were placed in an enriched Rat Park. Rats assigned to the Enriched Control (ENR) group received all of the same positive events (including items from the Rat Park) and the corresponding cues as the UPER group, but they received them all in their home cage at the same time each day to minimize anticipation throughout the day. Rats received either UPER or ENR training for 3 weeks, after which they underwent behavioral assessments prior to sacrifice for histological analyses. Image made using BioRender Software (BioRender.com).

Following transfer to their new enriched cages, each cage was randomly assigned to either an enhanced anticipation group (“cued Unpredictable Positive Event Response”/“UPER”) or an enriched control (“ENR”) group (Figure 1). For the enhanced anticipation UPER group, rats received three positive events randomly throughout the day (9.00 a.m.–5:00 p.m.), with each positive event acutely preceded by an associative cue to acutely enhance anticipation of receipt of the positive event. Thus, the chronic unpredictable presentation of positive events over a 24-h period, along with the short-term cued period, provided an enhanced anticipatory response for the UPER group; conversely, the ENR group experienced all three positive stimuli at a consistent, predictable time of day with no preceding associative cues---mitigating the anticipatory response. The cues and their associated positive events for the UPER group consisted of the following: (1) exposure to a Lego® block placed in the home cage for 15 min followed by administration of Froot Loops® treats in the home cages, (2) sunflower seeds with the shells on so that rats had an anticipatory tactile waiting period as they had to remove the seed shell prior to consumption, and (3) a three-minute anticipatory period in a transport cage prior to being placed in an enriched arena (“Rat Park,” 36″ L x 18” W x 17” H) for 7 min. The Rat Park arena consisted of aspen bedding, a plastic tunnel, a plastic structure the rats could enter, a red plastic running wheel, and several natural and artificial toys that were switched out each week. As previously mentioned, to enhance anticipation of time in the Rat Park, UPER rats were moved together with cage mates into a transfer cage and placed in a waiting context (similar to a waiting room) around which were several black and white images on the surrounding walls that served as associative contextual cues for the impending admittance to the Rat Park. A separate group of rats (Enriched “ENR” Controls) was used as a control group to control for receipt of positive events without enhanced anticipation. ENR rats received the same positive events (Froot Loops®, sunflower seeds with shell off, and objects from the Rat Park) plus the associated cues (i.e., Lego® block) simultaneously in their home cage every day at the same time (4.00–4:30 p.m.) to control for stimulus exposure while minimizing anticipation. Rats received either the anticipation-enhanced (UPER) or anticipation-minimized (ENR) positive events for 3 weeks, after which they underwent a series of behavioral assessments to determine the impact of chronic enhanced anticipation of positive events on cognitive strategies and stress responsiveness. Food was removed from rats’ home cages 3 h before the behavioral assessments so that animals were food restricted for the assessments.

### 2.3 Cognitive bias assessment

Each rat underwent a Cognitive Bias Assessment that consisted of three phases: a 2-day Habituation phase, a 3-day Cue Distinction Choice Training phase, and a one-day Ambiguous Cue Trial phase. However, before rats underwent the Cognitive Bias Assessment, their preference of Cheerio® and Froot Loop® was assessed to determine if their high-value (preferred) food was a Cheerio® or a Froot Loop®.

#### 2.3.1 Cheerio® versus Froot Loop® preference testing

To determine if rats preferred a Froot Loop® or a Cheerio®, three preference tests were administered, occurring 9, 4, and 1 day prior to the Cognitive Bias Assessment. For these three separate preference tests, rats were individually placed in a separate arena that contained both a Cheerio® and Froot Loop®. The first day of preference testing consisted of the Cheerio® and Froot Loop® being clearly presented and placed on the bedding of the arena. However, one rat (out of the twenty-three rats) did not engage in this task on the first day, so for the following two preference tests the Cheerio® and Froot Loop® were instead clearly and visibly elevated at rats’ eye level by two separate strings. Rats were allowed to investigate both the Cheerio® and Froot Loop®, and the first food they consumed was recorded. The choice of food that each rat ate 2 out of 3 times (or all 3 times) was labelled as the preferred “high-value” food.

#### 2.3.2 Habituation phase

**Figure 2:**
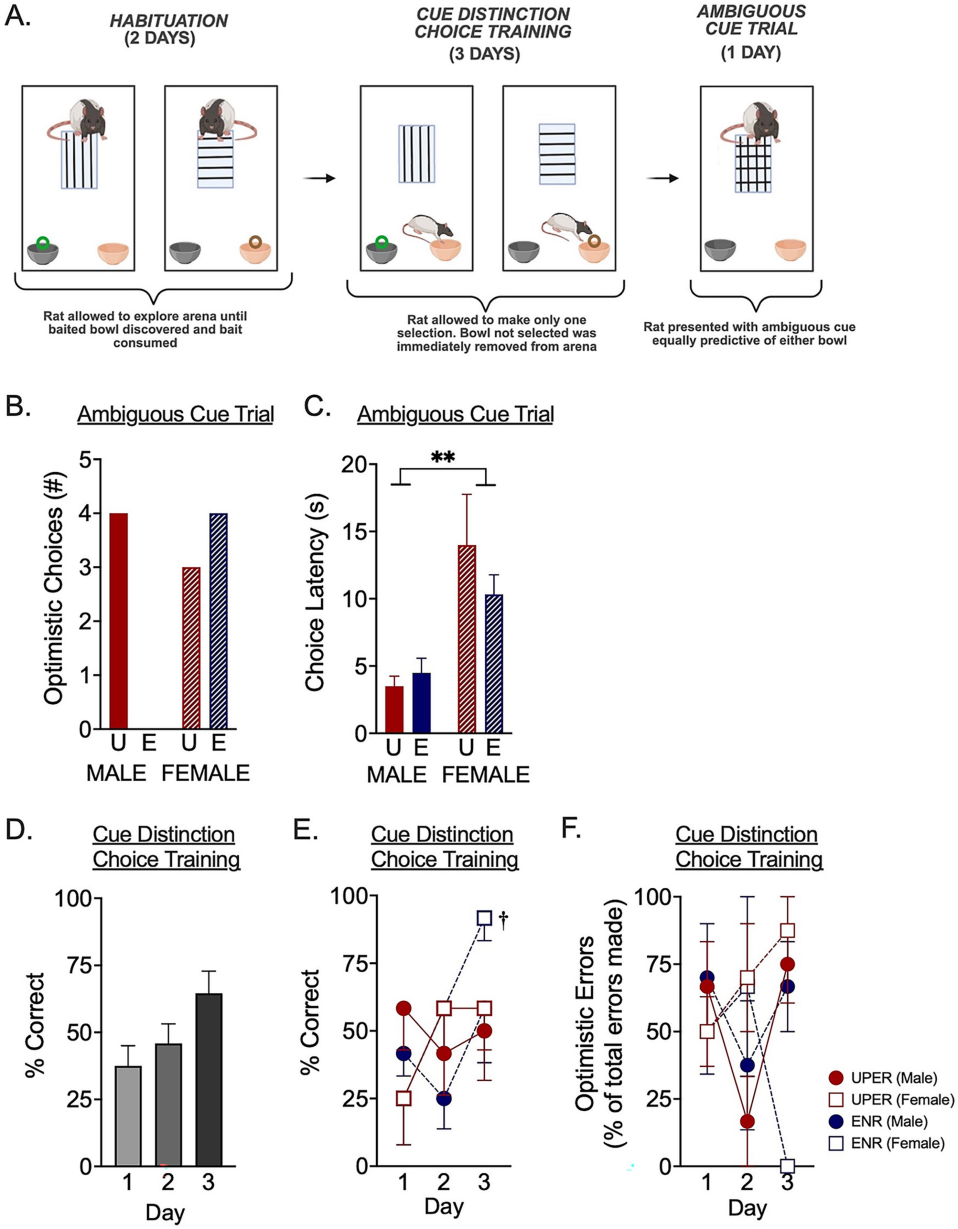
Cognitive bias assessment. **(A)** During the Habituation phase (days 1 and 2; **A**, left panel) of the cognitive bias assessment, rats were individually placed in an arena that contained one lined tile in the middle and two clay pots at one end. For two of the trials, a vertically lined tile was placed in the arena and a Froot Loop^®^ was placed in the black bowl. For the other two trials, a horizontally lined tile was placed in the arena and a Cheerio® was placed in the orange bowl. Rats allowed to explore the arena until they ate the Froot Loop^®^ or Cheerio^®^. Rats then advanced to Cue Distinction Choice Training phase on days 3–5 (**A**, middle panel). For Cue Distinction Choice Training, rats were individually placed in the same arena with the lined tile and baited bowl, but the rats allowed to make only one choice, as the unchosen bowl was removed from the arena following a choice. The Ambiguous Cue Trial phase occurred on day 6 to assess cognitive bias (**A**, right panel). Rats were individually placed in the arena and exposed to a tile that was both vertically and horizontally lined. The bowl they chose to investigate was recorded as either an Optimistic Choice (if the black bowl was chosen) or Pessimistic Choice (if orange bowl was chosen). **(B)** A Fisher’s exact test revealed a nonsignificant trend in different cognitive strategies in the Ambiguous Cue Trial (*p* = 0.0765). Specifically, UPER and ENR females made a similar number of optimistic choices in the face of an ambiguous cue; however, none of the ENR males selected a high-value optimistic choice compared to 66.7% of the UPER males selecting the high-value, optimistic choice when confronted with an ambiguous cue. **(C)** A 2 × 2 ANOVA revealed a main effect of sex for latency to make a choice in the Ambiguous Cue Trial, with females taking longer than males to make a decision in the face of an ambiguous cue. **(D)** A Friedman test comparing correct choices during Cue Distinction Choice Training over days 1–3, collapsed across training and sex, indicated a nonsignificant trend towards learning across the 3 days [*X*^2^(2) = 5.772, *p* = 0.056]. **(E)** A 3 × 2 × 2 mixed ANOVA to examine correct choices during Cue Distinction Choice Training revealed a significant interaction between day and sex driven by ENR females who exhibited near-perfect performance by the third day of training compared to the first day (^†^*p* = 0.0013 indicates results of Šídák’s multiple comparison’s test). **(F)** A 3 × 2 × 2 mixed ANOVA revealed a trend towards a training × day interaction for the number of optimistic errors on the final day of Cue Distinction Choice Training (*p* = 0.0537). Bars represent mean ± SEM. *p < 0.05; **p < 0.0. Image made using BioRender Software (BioRender.com).

To determine if enhanced anticipation training shapes cognitive bias, rats underwent a Cognitive Bias Assessment task (Figure 2) to determine if, in the final phase, they interpret an ambiguous cue as indicative of a positive outcome (i.e., optimistic cognitive strategy) or a negative outcome (i.e., pessimistic cognitive strategy). The Cognitive Bias Assessment involves initially training rats during a Habituation Phase for 2 days to associate a vertically lined tile with a highly desirable (“high-value”) Froot Loops® cereal piece in a black painted clay bowl and a horizontally lined tile with a less desirable (“low-value”) Cheerio® in an unpainted (orange) clay bowl (Figure 2A, left). For the Habituation Phase, rats were placed into an aspen bedding-lined arena (28″ L × 24” W × 20” H) containing both clay bowls (3.5″ diameter; 2.75″ high) at one end. When the vertically lined tile is presented in the arena, the high-value Froot Loop® is present in the black bowl (and the orange-colored Cheerio® bowl is empty). When the horizontally lined tile is present in the arena, a Cheerio® is present in the orange bowl (and the black Froot Loop® bowl is empty). On each of the 2 days, individual rats underwent four trials: for two of the trials, they were placed in the arena and presented with a vertically lined tile and the Froot Loop® in the black bowl and for two of the trials, they were placed in the arena and presented with a horizontally lined tile and a Cheerio® in the orange bowl. Rats were allowed to explore the arena until they discovered and ate the Froot Loop® or Cheerio® in the baited bowl and were removed as soon as they consumed the food. On the first day of Habituation, the first two trials consisted of a Froot Loop® in the black bowl and the second two trials consisted of a Cheerio® in an orange bowl. For the second day of Habituation, order presentation of the baited Froot Loop®/black bowl or baited Cheerio®/orange bowl was randomly picked to begin with the baited Cheerio®/orange bowl and proceeded to alternate between baited Cheerio®/orange bowl and baited Froot Loop®/black bowl.

#### 2.3.3 Cue distinction choice training phase

After 2 days of Habituation training, rats underwent 3 days of Cue Distinction Choice Training in which they were allowed to only select/investigate the first bowl they approached. This was to introduce the contingency that the high- or low-valued reward could only be retrieved from the first bowl that was approached and thus only one selection could be made (Figure 2A, middle). Hence, through the Cue Distinction Choice Training phase, rats learned that there was a consequence for choosing the wrong bowl first---i.e., they were only allowed to consume the cereal piece in the bowl that was initially selected. To establish this first-bowl/reward choice contingency, on each day of Cue Distinction Choice Training, rats underwent the same task as Habituation in that rats were placed in the arena and presented with either the vertically or horizontally lined tile and allowed to explore for 2 min. However, in these trials, the non-selected bowl was removed after the rats made their initial choice. If rats were presented with a vertically lined tile and chose the orange (empty) Cheerio® bowl, the black (baited) Froot Loop® bowl was then removed. If rats were presented with the horizontally lined tile and chose the black (empty) Froot Loop® bowl first, then the orange (baited) Cheerio® bowl was removed from the arena. These trials established the contingency that rewards were only retrieved from the first bowl that was selected. Each rat underwent four trials on days 1 and 2 of the Cue Distinction Choice Training Trials and two trials on day 3. Because rats were exposed to more trials on days 1 and 2, only the first two trials each day were scored for analysis of trial performance across days. Performance was calculated as percent of choices made that were the correctly cued baited bowl out of total trials attempted. Errors were assessed and scored as either optimistically-skewed or pessimistically-skewed errors based on the following criteria: errors made when a rat was exposed to a horizontal line (indicating a Cheerio® in the orange bowl) but chose the black Froot Loop® bowl were scored as optimistically-biased errors. Similarly, errors made when rats were exposed to a vertical line (indicating a Froot Loop® in the black bowl) and chose the orange-colored Cheerio® bowl were scored as pessimistically-skewed errors. Percent optimistic or pessimistic errors were calculated as the percent of errors that were either optimistic or pessimistic out of the total errors made.

#### 2.3.4 Ambiguous cue trial

Following the 3 days of Cue Distinction Choice Training, rats underwent the Ambiguous Cue Trial phase to determine the presence of either an optimistic or pessimistic cognitive bias in response to an ambiguous cue that was equally predictive of a baited Cheerio® bowl and Froot Loop® bowl (Figure 2A, right). Each rat underwent a single trial in which they were presented with a tile that had *both* horizontal and vertical lines so that it was equally predictive of either a high-value (cued by vertical lines) or low-value (cued by horizontal lines) bowl. In this trial, rats were allowed to select either the black bowl or the orange bowl. Both bowls were unbaited to reduce olfactory cues associated with Froot Loops® and Cheerios® cereal pieces. The latency to choose a bowl was determined as the time from the start of the trial until the rat investigated a bowl’s contents and placed its entire snout fully down into the bowl. Bowl choice was recorded as either a high-value “Optimistic Choice” (if they chose the black bowl) or a low-value “Pessimistic Choice” (if they chose the orange bowl), with a high-value choice interpreted as an optimistic cognitive bias and a low-value choice interpreted as a pessimistic cognitive bias. The total number of optimistic choices made for each group (UPER and ENR males and females) was recorded.

### 2.4 Novelty-suppressed feeding task

**Figure 3:**
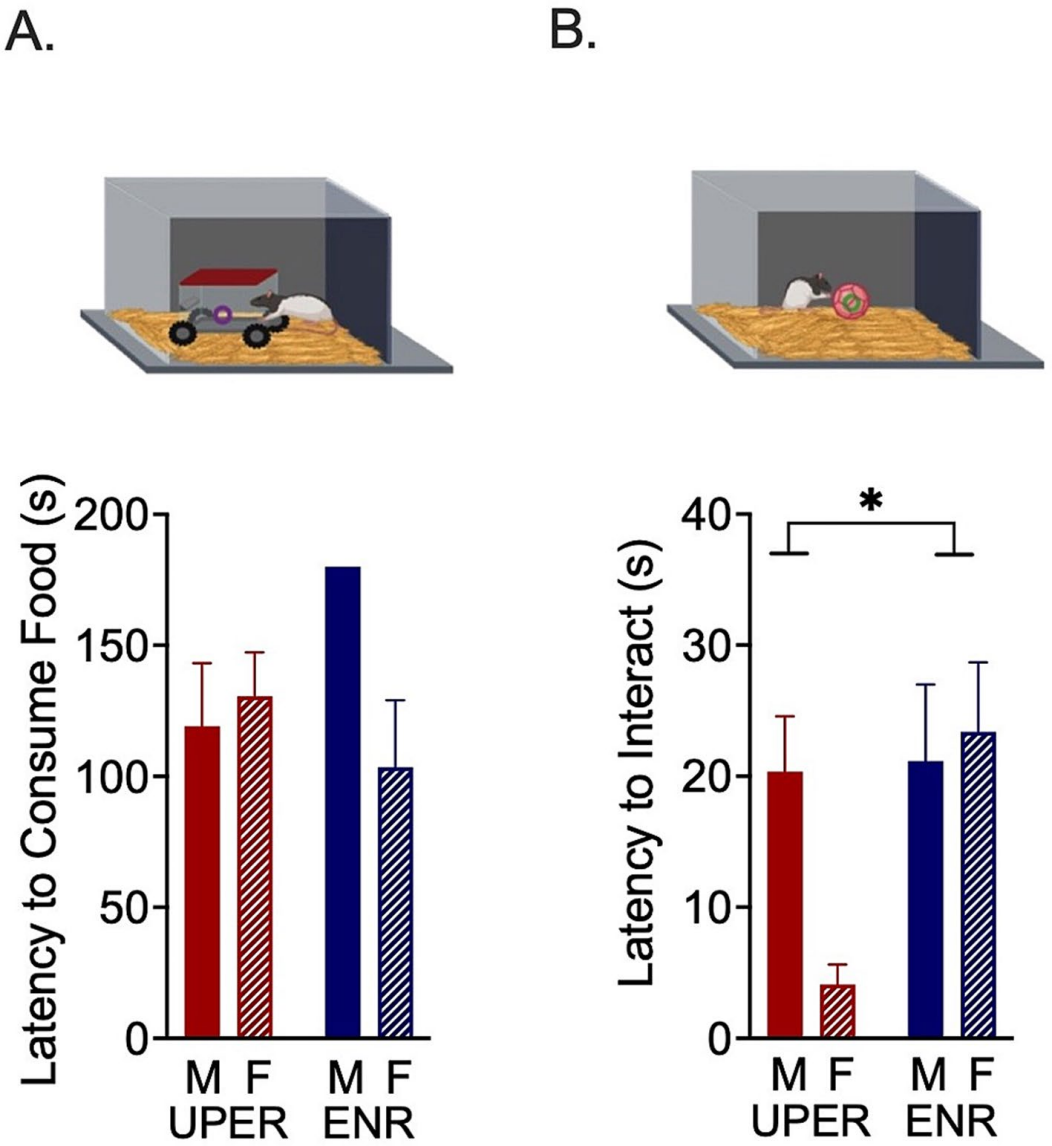
Novelty-suppressed feeding task and unattainable puzzle reward task. **(A)** A 2 × 2 ANOVA revealed a significant training by sex interaction on latency to consume a Froot Loop^®^ in the novel environment. None of the ENR males consumed the FrootLoop^®^ in the novel environment compared to ENR females (posthoc revealed nonsignificant trend between ENR males and ENR females, *p* = 0.0773). **(B)** A significant main effect of training was seen in the Unattainable Puzzle Reward Task, with UPER rats approaching the novel object more quickly, largely driven by UPER female rats’ quicker approach. Bars represent mean ± SEM. *p < 0.05; **p < 0.01.

In the afternoon following the Ambiguous Cue Trial phase, all rats underwent a Novelty-Suppressed Feeding Task (Figure 3A). Rats were individually placed in a novel open arena (27.5 cm x 27.5 cm) containing corncob bedding that was redistributed between animals to distribute the previous animal’s scent. A novel object (a rodent-sized model car with a covered cabin accessible from one side) was placed in the middle of the arena. A piece of Froot Loop® was placed inside the car and rats were allowed to explore the arena and car for 3 min. The latency to interact with the car/novel object, total number of interactions, interaction duration, and latency to consume the food inside the car were recorded. If rats ate the Froot Loop®, they were allowed to finish consuming the treat and then removed from the arena and the trial was concluded. If rats never ate the Froot Loop®, a time of 180 s was recorded. Trials were recorded using Noldus software (Noldus, Leesburg, VA, USA) and the number of visits to the novel object, latency to approach the novel object, percent of time spent exploring the novel object, and latency to eat the Froot Loop® were scored by an experimenter blinded to treatment group.

### 2.5 Unattainable puzzle reward task

Twenty-four hours following Novelty-Suppressed Feeding Task, persistency of each rat was assessed in an Unattainable Puzzle Reward Task in which rats were exposed to an unsolvable puzzle and attempted to retrieve an unattainable Froot Loop® reward (Figure 3B). Specifically, rats were individually placed in the same arena that had previously contained the novel object but this time the arena was empty with no car/novel object. A plastic ball marketed as a cat toy (Wonpet Co., Ltd., Guangzhou, China) and containing a Froot Loop® inside was placed in the middle of the arena. Because the ball had small holes in it, the rats could see, smell, and occasionally try to touch the Froot Loop® but could not remove it from the ball. Rats were allowed to explore the arena and try to retrieve the Froot Loop® for 3 min. The latency to interact with the ball was recorded by Noldus software (Noldus, Leesburg, VA, USA) and the duration and number of interaction bouts were scored by experimenters blinded to experimental group assignments.

### 2.6 Neuronal activation during anticipation

To determine which brain regions were active during anticipation of an appetitive event, 72 h after the Unattainable Puzzle Reward task, rats were individually exposed to a Lego® block 90 min prior to sacrifice. To ensure that all rats were perfused at the time of optimal c-fos expression, it was necessary to expose rats individually to the Lego® block every 15 min, as perfusions were all approximately 15 min apart. Thus, rats were individually placed in small transfer cages lined with aspen bedding and containing a Lego® block and remained in the transfer cage for 15 min to activate the anticipatory response. After 15 min, rats were returned to their enriched home cages where they remained until they were sacrificed 90 min after exposure to the Lego® Block. Subsequently, brains were harvested and processed for immunostaining.

### 2.7 Immunostaining, microscopy and analysis

Ninety minutes after Lego® block exposure, rats were deeply anesthetized with isoflurane and transcardially perfused with 200 mL PBS followed by 200 mL 4% paraformaldehyde. Brains were extracted and stored at 4 °C in 4% paraformaldehyde for post-fixation. Coronal sections 40 μm thick containing the nucleus accumbens (from Bregma: 2.28 to 0.48 mm AP) and the hypothalamus (paraventricular nucleus (PVN) and the perifornical area (PeFLH); from Bregma: −1.92 to −3.72 mm AP) were cut at −25 °C on a cryostat (Thermo-Fisher Scientific, Waltham, MA, United States). To avoid duplicate sections from being quantified, every sixth section was kept, allowing for 240 μm between each section for analysis.

For c-fos immunolabeling, free-floating sections were incubated in PBS containing 0.3% H_2_0_2_ then blocked for 60 min at room temperature in PBS containing 0.3% Triton X-100, 0.1% bovine serum albumin, and 10% normal goat serum. Sections were then incubated with a rabbit anti-cfos antibody (1:5,000; Immunostar Cat #26209) in PBS containing 0.3% Triton X-100, 0.1% bovine serum albumin, and 5% normal goat serum for 48 h rocking at 4 °C followed by incubation with a biotinylated goat anti-rabbit secondary antibody (1:500; Vector Laboratories Cat# BA-1000) in PBS containing 0.3% Triton X-100, 0.1% bovine serum albumin, and 1% normal goat serum for 2 h rocking at room temperature. Following a two-hour incubation in Vectastain Elite ABC Solution (Vector Laboratories, Burlington, VT, USA), sections were incubated in PBS containing 0.6% Tris buffer, 0.3% NH_3_Nis, 0.02% diaminobenzidine (DAB; Sigma-Aldrich, St. Louis, MO, USA) and staining was subsequently developed with 0.6% H_2_0_2_. Sections were mounted onto gelatinized slides and coverslipped using Permount mounting media (Electron Microscopy Sciences, Hatfield, PA, USA).

C-fos immunolabeled (c-fos+) cells were visualized and imaged at 40× using a Keyence BZ-X800 All-In-One Fluorescent Microscope. Bilateral images were taken 500 μm lateral to the 3^rd^ ventricle (PVN region) and 1,500 μm lateral to the 3^rd^ ventricle (PeFLH). Images were then exported into ImageJ (FIJI) and the number of c-fos + positive cells for each region was manually counted by an investigator blinded to the experimental groups of each animal. The total number of immunopositive cells from each image was averaged to get the mean number of c-fos + cells per visual field for each animal.

### 2.8 Endocrine responses

Corticosterone (CORT) levels were assessed from fecal samples taken at baseline prior to onset of UPER or ENR training (after habituation to home cages and immediately prior to transfer to enriched cages). Post-UPER and post-ENR samples were taken after the last week of UPER/ENR training (specifically 72 h after the last behavioral assessment to reduce the influence of the behavioral assessments on any potential CORT responses). To collect fecal samples from each rat and to avoid contamination of the samples, rats were temporarily placed in individual standard transport cages and allowed to pass a fecal bolus as normal before being placed back in their home cage. The collected samples were stored at −80 °C until analyzed. For analysis, samples were thawed and hormones levels were assessed and quantified using an ELISA kit (Enzo Life Sciences, Farmingdale, NY, United States) following methods previously validated (Kent et al., 2022). Optical densities of samples were read using an automated microplate reader (BioTek, Winooski, VT, United States) and Gen5 software (Version 2.04.11; BioTek, Winooski, VT, United States). The intra- and inter-assay coefficients of variance for the CORT assay were 6.6 and 7.8%, respectively.

### 2.9 Data analysis and statistics

Data were analyzed in GraphPad Prism (Version 10; GraphPad Software). Differences between groups were compared using a Friedman Test with post-hoc Šídák’s multiple comparison’s test (Cue Distinction Choice Training; when not normally distributed), a 2 × 2 ANOVA with post-hoc Tukey test (Ambiguous Cue Trial latency; novelty-suppressed feeding behaviors; Unattainable Puzzle Reward test behaviors; histology), a two-way ANCOVA (endocrine analysis), 3 × 2 × 2 mixed ANOVA (group differences in Cue Distinction Choice Training over time; group differences in error bias in Cue Distinction Choice Training over time), and Fisher’s Exact Test (Ambiguous Cue Trial). For all analyses, if a data point met the criteria for a statistical outlier per Grubb’s test, it was removed.

## 3 Results

### 3.1 Cognitive bias assessment

Prior to the Cognitive Bias Assessment, each rat’s preference for either a Cheerio® or FrootLoop® was determined. Of the twenty-four rats, twenty-three demonstrated preference for a FrootLoop® as indicated by choosing to consume a FrootLoop® instead of a Cheerio® on two or all three of the three preference tests. One rat, however, did not engage in the task on the first day of preference testing and therefore did not eat either a Cheerio® or FrootLoop®. Thus, the criteria to analyze the rat’s latency to forage for and choose a food choice was utilized and it was observed that this rat preferred FrootLoops® based on the decreased latency to select a FrootLoop® over a Cheerio® in 100% of the assessments in which both food choices were presented to the rat.

Following anticipation enhanced (UPER) and anticipation minimized (ENR) training, a nonsignificant sex-dependent effect was observed in the Ambiguous Cue Trial phase of the Cognitive Bias Assessment (Figure 2B). A Fisher’s exact test was used to determine if there was an association between the training group and high-value choice (*p* = 0.076) and revealed a nonsignificant trend in sex differences in baseline optimism, with anticipation-enhanced training increasing optimistic responses in males (Figure 2B). No differences were observed between the UPER- and ENR-trained female rats. When examining the latency to respond, a 2 × 2 ANOVA revealed a main effect of sex, with females taking longer than males to choose a bowl following the presentation of the ambiguous cue (Figure 2C; [*F*(1,20) = 14.83; *p* = 0.001; ηρ^2^ = 0.426].

To assess rats’ ability to learn the associations between the visual cues and baited bowls, correct choices made during the 3 days of Cue Distinction Choice Training were analyzed to determine if rats were correctly associating the vertically-lined and horizontally-lined tiles with the Froot Loop®-baited or Cheerio®-baited bowls, respectively. Overall, correct responses improved by 27.1% over the 3 days of training, and a Friedman test revealed a nonsignificant trend towards learning to correctly discriminate between the two visual cues predicting either a Froot Loop® -baited or Cheerio®-baited bowl over the 3 days of learning (Figure 2D; [*X*^2^(2) = 5.772, *p* = 0.056]. However, when examining how each individual group (male and female UPERs and ENR) learned to discriminate the tiles over the 3 days of Cue Distinction Choice Training using a 3 × 2 × 2 mixed ANOVA, a significant interaction between day and sex was found [*F*(2,40) = 3.595; *p* = 0.0367; ηρ^2^ = 0.152], driven by increased learning in ENR females between days 1 and 3 (Figure 2E). Interestingly, this increased evidence of accurate bowl selection over 3 days in the Cue Distinction Choice Training trial was due to nearly perfect performance on the task by the ENR females, as this group identified the correct bowl 91.67% of the time following the tile presentations. In contrast, UPER females only made the correct choice 58.3% of the time. Thus, although individual and group differences were observed, it is important to acknowledge that there appeared to be differences in successful learning between the groups.

Given the relevance of error bias in the context of optimistic cognitive strategies, error biases during Cue Distinction Choice Training were then assessed and scored as either optimistically-skewed or pessimistically-skewed errors. Errors made when a rat was exposed to a horizontal line but incorrectly chose the bowl associated with a high-value Froot Loop® were scored as optimistically-biased errors. Similarly, errors made when rats were exposed to a vertical line but instead chose the low-value cheerio®-associated bowl were scored as pessimistically-skewed errors. Interestingly, by the third day of training, when three out of the four groups made errors, a majority of their errors were optimistic errors except for ENR females who made no optimistic errors, as a 3 × 2 × 2 mixed ANOVA revealed a nonsignificant trend towards an interaction between day and training with a moderate effect size [Figure 2F; *F*(2,34) = 3.192; *p* = 0.054; ηρ^2^ = 0.158]. Specifically, whereas both UPER and ENR males demonstrated similarly high levels of optimistic errors, ENR females exhibited fewer optimistic errors.

### 3.2 Novelty-suppressed feeding task

To determine hyponeophagia, rats were placed in a novel arena that contained a novel object (a model car) with a Froot Loop® inside. Interaction with the car, approach latency, total interaction duration (seconds), interaction bouts and latency to consume the Froot Loop® were recorded and analyzed. No differences were observed between UPER and ENR groups in latency (s) to approach the car, total time (s) spent exploring the car, or bouts of exploration of the car, suggesting that UPER training did not affect exploratory behavior. When examining latency to consume a Froot Loop® inside the car (a novel object), a 2 × 2 ANOVA revealed a significant interaction between training and sex [*F*(1,18) = 4.885 *p* = 0.0403; ηρ^2^ = 0.213; *n* = 6 per male/female UPER group and *n* = 5 per male/female ENR group due to one statistical outlier and one animal who was excluded due to experimenter error during trial]; a posthoc analysis revealed a nonsignificant trend characterized by the ENR males taking longer to consume the Froot Loop® than ENR females (Figure 3A; *p* = 0.0773) with no differences observed between the UPER groups.

### 3.3 Unattainable puzzle reward task

In the Unattainable Puzzle Reward Task, the behavioral task examining persistence and latency (s) to interact with a novel object in a familiar arena, the total duration (s) spent interacting with the ball and the number of interactions with the ball were recorded. A significant main effect of training on latency to interact with the ball was observed [Figure 3B; *F*(1,19) = 4.469; *p* = 0.0480; ηρ^2^ = 0.190; *n* = 6 per group and *n* = 5 in UPER female due to statistical outlier]. No differences in interaction bouts or duration between UPER and ENR groups were found, as well as no differences in the time spent in the center of the arena.

### 3.4 Neuronal and endocrine activation during anticipation

**Figure 4:**
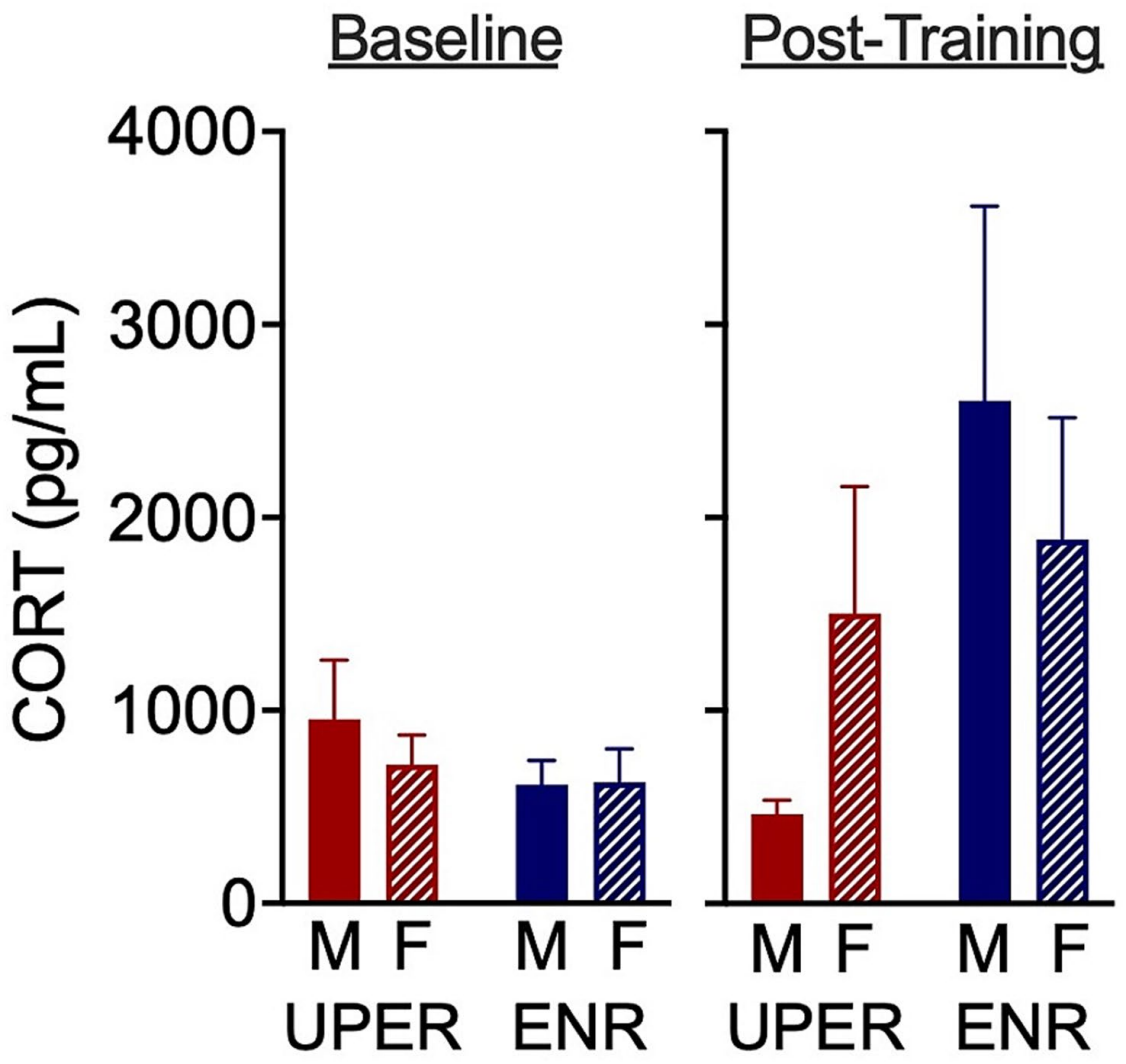
Fecal corticosterone metabolites following UPER or ENR training. A two-way ANCOVA revealed no significant sex x training interaction on post-training CORT levels [with baseline (pre-training) levels used as a covariate]. However, a nonsignificant trend for training was observed, with UPER rats exhibiting lower CORT levels than ENR rats [*F*(1,15) = 3.49, *p* = 0.08]. Bars represent mean ± SEM.

In a task intended to determine neuronal activation during anticipation in response to a Lego® Block, robust c-fos expression was observed restricted to the paraventricular nucleus of the hypothalamus (PVN) and the perifornical area in the posterior lateral hypothalamus (PeFLH). Neither training nor sex had an effect on PVN or PeFLH activation during the Lego® Block exposure. Extremely faint but barely detectable c-fos expression was also seen in the piriform cortex, somatosensory cortex and basal forebrain, suggesting putative mild neuronal activation in these areas (data not quantified). In examining fecal corticosterone metabolites following UPER or ENR training, a two-way ANCOVA revealed no significant sex x training interaction on post-training CORT levels using baseline (pre-training) CORT levels used as a covariate, though a nonsignificant trend for training was observed with UPER rats displaying lower CORT levels than ENR rats [*F*(1,15) = 3.49; *p* = 0.08; *n* = 3–6 per group); ηρ^2^ = 0.189; Figure 4].

## 4 Discussion

The current study introduces a novel rodent model for examining the neurobiological effects of chronic anticipation of positive events. Specifically, we assessed the hypothesis that chronic anticipation of positive events would shift cognitive bias, persistence, and novelty-suppressed feeding towards a more optimistic, persistent, and anxiolytic phenotype. Findings from the current study suggest that 3 weeks of chronic anticipation of positive events (UPER training) administered randomly throughout the day produced sex-dependent effects across cognitive, behavioral, and affective domains. Whereas UPER training resulted in a nonsignificant trend in a shift away from the pessimistic cognitive bias seen in ENR males that received no positive anticipation training, females’ cognitive bias was not as affected by UPER training. Data from the Novelty-Suppressed Feeding Task indicated an interaction between UPER training and sex in hyponeophagic-related behavior. Although males exhibited higher latencies to consume the food in the ENR group, the UPER males’ latencies did not differ from the UPER females. Interestingly, in the Unattainable Puzzle Reward Task, a training effect was observed with the UPER rats displaying a decreased latency to interact with a novel object than the ENR rats. Thus, although the UPER training resulted in sex-dependent effects, evidence of behavioral differences was apparent for both males and females in a task-dependent manner.

Previous studies in humans have reported that the anticipation of positive events, distinct from the positive event itself, intensifies the perceived emotional response to the thought of that event in addition to conferring resiliency to a life stressor (Leslie-Miller et al., 2021; Monfort et al., 2015). Given that compromises in positive anticipation have been associated with mental illnesses such as depression (Sherdell et al., 2012), determining the lifelong impact of positive anticipation may reveal novel strategies for preventing psychiatric symptoms. A recent study provided children diagnosed with cancer the ability to make, and therefore anticipate, a wish of their choice (Shoshani et al., 2016). Highlighting the powerful role that anticipation of positive events can have on mental health, the authors found that the children who were randomly assigned to the Make-A-Wish group, and thus able to anticipate their wish coming true, demonstrated reduced distress and improved mental health outcomes compared to the control group who were not provided with the opportunity to make a wish (Shoshani et al., 2016).

In the current study, UPER training consisted of layering acute anticipation of positive events (due to a conditioned stimulus like a Lego® Block associated with receipt of an unconditioned reward 15 min later) onto a longer temporal scale (due to the randomization throughout the day of the multiple positive events). Specifically, the protocol layered acute anticipation of positive events onto a randomized daily schedule. Three distinct cues were used: a Lego® block (visual), sunflower seeds (tactile), and a transfer cage to a rat park (contextual). Informal observations of the UPER rats’ responses to the experimenters entering the lab (e.g., approaching the cage wall closest to the experimenter) provided anecdotal evidence of intensified anticipation as the UPER training progressed. Future studies should consider recording and analysis of ultrasonic vocalizations (USVs) emitted during these anticipatory periods as another indicator of the affective state of UPER rats, as emission of USVs in various frequency ranges has been shown to reflect the emotional state of rodents (Simola and Granon, 2019).

In humans, a negative cognitive bias is more frequently reported in both healthy and depressed females compared to males (Kinari, 2016; Mansour et al., 2006; Rajdev and Raninga, 2016). Whereas previous studies in both humans and rodents have reported more prevalent optimistic cognitive strategies in males than females, the results of the current study suggested that males displayed lower levels of baseline optimism. However, it is worth noting that many animal and human studies observing male and female differences in optimism refer to how they process *risk,* with males tending to be less risk averse than females (for a review see Orsini et al., 2022). Accordingly, risk assessment may represent a potential underlying neural mechanism contributing to sex differences in optimistic strategies (Dohmen et al., 2023). As the Cognitive Bias Assessment in this study utilized a high-value food reward and a low-value food reward, the risks were relatively low. Interestingly, the results of the current study’s relatively low-risk Ambiguous Cue Trial phase of the Cognitive Bias Assessment indicate that after UPER training, males shifted to a more optimistic cognitive profile in the presence of an ambiguous cue. Given the known sex differences in psychiatric disorders like anxiety and depression (for a review see Bangasser and Cuarenta, 2021), the sex-specific effects of UPER training on cognitive strategies have implications for preventive therapies.

Notably, in the current study, there were group-dependent outcomes in the Cue Distinction Choice Training phase, with the ENR females demonstrating the most proficiency. Future studies should consider lengthening Cue Distinction Choice Training by a few days to further explore this observed difference in learning in ENR females. In the current study, we refrained from excluding animals from the Ambiguous Cue Trial phase to avoid biasing the assessment towards high learners (Krakenberg et al., 2019a). Another consideration when designing the Ambiguous Cue Trial phase to assess cognitive bias is the value of a cued reward (appetitive outcome) versus punishment (aversive outcome), as an option assess cognitive bias. Such a paradigm presents one stimulus that cues an appetitive outcome and another stimulus that cues an aversive outcome, with animals being allowed to press a lever in response to the first cue to receive the reward and press another level in response to the latter cue to avoid an aversive punishment (Lagisz et al., 2020; Saito et al., 2016). Including an appetitive and aversive contingency choice may allow rats to more quickly learn the task. Finally, the choice of sensory modality for the cognitive bias assessment should be strongly considered, as previous research indicates a difference in baseline optimistic or negative cognitive bias depending on the sensory modality and the affective state of the animal; for example, tasks utilizing auditory and tactile cues largely find a pessimistic bias when animals are exposed to negative environments and optimistic bias when animals are exposed to positive environments (for a review see Lagisz et al., 2020; Nguyen et al., 2020). Whereas cognitive bias tasks have frequently utilized auditory and tactile cues, very few have utilized visual cues to predict outcomes in cognitive bias assessment tasks, likely due to the added challenges that rodents experience in visual cue discrimination tasks (Krakenberg et al., 2019b; Nguyen et al., 2020).

In the Novelty-Suppressed Feeding Task, reduced exploration, as well as hesitation to consume food in a new environment, are viewed as indicators of depression- and anxiety-like behavior in preclinical animal models, with chronic antidepressant use reversing feeding inhibition in a new environment (Gencturk and Unal, 2024; Nestler and Hyman, 2010; Samuels and Hen, 2011). Studies that have examined how context affects feeding behaviors report that females tend to be affected by, and discriminate between, new contexts more than males (Greiner et al., 2024; Greiner and Petrovich, 2020; Reppucci et al., 2013). Our findings that ENR females consumed food in a novel environment more quickly than ENR males, contradicts the previously mentioned findings. The sex-by-training interaction observed in the Novelty-Suppressed Feeding Task in the current study suggests that UPER training in males leads to a shift in optimistic cognitive strategies in this task as well as the Cognitive Bias Assessment, as UPER males were less hesitant to consume food in the novel environment compared to their untrained ENR counterparts. Given that exploration hesitancy in novel environments is a predictor of vulnerability to depressive symptoms in rodent models (Stedenfeld et al., 2011), these findings emphasize the relevance of enhanced anticipation training for preventative and therapeutic interventions. The observation of sex-dependent effects of UPER training provides further motivation to use this preclinical model of enhanced anticipation training in future studies that incorporate preclinical models of disease, given reported sex-dependent effects in emotional disorder phenotypes in humans (for reviews see Eid et al., 2019; Ramikie and Ressler, 2018).

Few human and rodent studies have examined the effects of positive events on cortisol and corticosterone levels, respectively. Results from human studies also suggest that positive affect is associated with reduced cortisol levels (Bostock et al., 2011; Nicolson et al., 2020; Pluess et al., 2012; Steptoe et al., 2005), though one study reported no association (Peeters et al., 2003). However, these studies examined associations of positive events and positive emotion with cortisol levels instead of cued positive events that were anticipated. To our knowledge, this is the first study examining anticipation of positive events that utilizes acute (minutes), daily (24 h) and chronic (3 weeks) positive stimuli exposure. A nonsignificant trend that UPER-trained rats had lower fecal corticosterone levels than their enriched control counterparts suggests that chronic anticipation of positive events may confer a buffering effect to elevated cortisol levels. This interpretation was strengthened by an effect size of 0.189. Because rodent studies have shown that females tend to have a higher stress-induced increase in corticosterone than males, it would be of interest to determine the effects of enhanced anticipation on stress-induced corticosterone in UPER versus ENR rats (Lu et al., 2015; Nalepa et al., 2025). Given the complex findings in humans indicating that stress-elicited cortisol increase is different in males and females depending on the modality of the stressor, it’s important to consider specific characteristics of the stress modality into when determining the effect of UPER and ENR training on cortisol levels in humans (Henze et al., 2021; Liu et al., 2017; Reschke-Hernández et al., 2017; Zimmer et al., 2003).

While distinct clusters of robustly-labeled c-fos + neurons were observed in the paraventricular nucleus of the hypothalamus (PVN) and the perifornical area in the lateral hypothalamus (PeFLH) during anticipation of a positive event, no training effects were observed. However, the PVN and PeFLH consists of a diverse array of neuronal diversity, and the robust activation of neurons within the PVN may reflect the activity of multiple cellular types, such as oxytocinergic neurons (Berkhout et al., 2024; Bonnavion et al., 2016; Kania et al., 2020). We are currently investigating specific types of neurons in the PVN, based on neurochemical characteristics, in UPER-trained animals compared to controls.

There are methodological challenges inherent to the time-window required for c-fos expression that make it difficult to fully attribute c-fos activation to anticipation. First, throughout the UPER training period, UPER-trained rats were exposed to a Lego block followed by receipt of a Froot Loop treat 15 min later. Prior to sacrifice, all rats were exposed to a Lego block for 15 min later, after which it was removed from their cage. As rats were sacrificed 90 min after Lego block exposure, the c-fos expression could be reflective of the 15-min anticipation window (in the UPERs), or it could be reflective of frustration after the Lego block was removed.

A few limitations of the current study should be noted. Although the ENR-trained groups served as a control for positive events anticipation experienced in the UPER-trained rats, the ENR rats experienced a limited and restricted anticipatory response when all positive events were experienced at a single time each day. Accordingly, a control group with no positive experiences each day would be informative in future investigations of the UPER paradigm. Additionally, given the impact of physical activity on cognitive and emotional responses (Gaertner et al., 2018; Giles et al., 2018; Hötting and Röder, 2013; Pearce et al., 2022; Wu et al., 2022), future investigations should include a running wheel in the home cages of all animals, regardless of the training group, so that all rats have access to this form of physical activity. In the current study, the UPER group-housed rats had minimal access to the running wheel (i.e., 7 min per day) but access for all animals is important as the impact of UPER training is investigated.

The UPER training protocol represents a novel rodent model of increased anticipation of positive events and its implementation to shape future behaviors. While previous studies have examined the impact of positive events, demonstrating the benefits they confer for mental health (Beevers and Meyer, 2002; Disabato et al., 2017; Hovenkamp-Hermelink et al., 2019; Lewinsohn and Graf, 1973; Lewinsohn and Libet, 1972; Spinhoven et al., 2011), to our knowledge this study is the first preclinical model that provides an opportunity to systematically examine the neurobiological impact of extended anticipatory training in rodents. Preclinical models of anticipatory training, such as the UPER model investigated in the current study, have the potential to advance behavior-based treatments for mental health disorders, offering innovative strategies for the development of behavior-based therapeutic approaches.

## Data Availability

The raw data supporting the conclusions of this article will be made available by the authors, without undue reservation.
